# Electronic forms for patient reported outcome measures (PROMs) are an effective, time-efficient, and cost-minimizing alternative to paper forms

**DOI:** 10.1186/s12969-021-00551-z

**Published:** 2021-05-03

**Authors:** Jennifer Y. Yu, Talia Goldberg, Nicholas Lao, Brian M. Feldman, Y. Ingrid Goh

**Affiliations:** 1 The Hospital for Sick Children, 555 University Avenue, Toronto, Ontario M5G 1X8 Canada; 2 Child Health Evaluative Sciences, SickKids Research Institute, Toronto, Ontario Canada; 3 Department of Pediatrics, Faculty of Medicine, University of Toronto, Toronto, Ontario Canada; 4 Institute of Health Policy, Management and Evaluation, Dalla Lana School of Public Health, University of Toronto, Toronto, Ontario Canada

**Keywords:** Patient reported outcome measures (PROMs), Childhood Health Assessment Questionnaire (CHAQ), Quality of my life (QoML), Technology, Electronic form, Digital form, Effectiveness, Equivalence, Acceptability, Comparison

## Abstract

**Background:**

Patient reported outcome measures (PROMs) provide valuable insight on patients’ well-being and facilitates communication between healthcare providers and their patients. The increased integration of the technology within the healthcare setting presents the opportunity to collect PROMs electronically, rather than on paper. The Childhood Health Assessment Questionnaire (CHAQ) and Quality of My Life (QoML) are common PROMs collected from pediatric rheumatology patients. The objectives of this study are to (a) determine the equivalence of the paper and electronic forms (e-form) of CHAQ and QoML questionnaires; (b) identify potential benefits and barriers associated with using an e-form to capture PROMs; and (c) gather feedback on user experience.

**Methods:**

Participants completed both a paper and an e-form of the questionnaires in a randomized order, following which they completed a feedback survey. Agreement of the scores between the forms were statistically analyzed using the intraclass correlation coefficient (ICC) (95 % Confidence Interval (CI)) and bias was assessed using a Bland-Altman plot. Completion and processing times of the forms were compared using mean and median measures. Quantitative analysis was performed to assess user experience ratings, while comments were qualitatively analyzed to identify important themes.

**Results:**

196 patients participated in this project. Scores on the forms had high ICC agreement > 0.9. New patients took longer than returning patients to complete the forms. Overall, the e-form was completed and processed in a shorter amount of time than the paper form. 83 % of survey respondents indicated that they either preferred the e-form or had no preference. Approximately 10 % of respondents suggested improvements to improve the user interface.

**Conclusions:**

E-forms collect comparable information in an efficient manner to paper forms. Given that patients and caregivers indicated they preferred completing PROMs in this manner, we will implement their suggested changes and incorporate e-forms as standard practice for PROMs collection in our pediatric rheumatology clinic.

## Background

Patient reported outcome measures (PROMs) provide valuable insight into patients’ experiences and are an important part of high-quality healthcare. Used in clinical and research settings, these standardized, validated questionnaires measure the patients’ perception (without other’s influences) of their condition and the impact of healthcare interventions [1, 2]. PROMs often ask patients to self-report general well-being and quality of life, symptoms, functional status, and condition-specific outcomes [1]. Integrating PROMs into practice facilitates better communication and engagement between healthcare providers and their patients, creating an environment where patients feel more comfortable with disclosing detailed information about their health status [3, 4]. This is important because although healthcare providers have tools to objectively measure the state of medical conditions, there are some subjective measures that can only be assessed by the patient.

In pediatric rheumatology, two commonly used PROMs are the Childhood Health Assessment Questionnaire (CHAQ) and the Quality of My Life (QoML) [5]. These PROMs can be completed by the patient or using a caregiver as a proxy [6, 7].

The CHAQ assesses outcome dimensions of disability, and pain and discomfort [8]. It is a widely used and well-validated measure that has been translated into numerous languages [9–12]. The CHAQ assesses physical function by asking a series of questions on a scale of 0 (no difficulty) to 3 (unable to do) about patients’ ability to perform activities of daily living (ADL) including dressing and grooming, arising, eating, walking, hygiene, reaching, gripping and activities [8, 13].

The QoML questionnaire is a validated tool which assesses the patient’s quality of life (overall and health-related) [7, 14, 15]. Patients are asked to indicate on a 10-cm horizontal-anchored visual analog scale (VAS) (where 0 = worst and 100 = best): 1) their overall quality of life and 2) their health-related quality of life [15].

All patients/caregivers attending the rheumatology clinic at The Hospital for Sick Children (SickKids) complete a standardized four-page paper form containing the original version of the CHAQ (which contains the additional VAS question “how would you rate your child’s illness in the past week”) and QoML questionnaires at each of their visits. This has been the standard practice in the clinic for about 25 years. There are, however, numerous problems associated with paper versions of PROMs which have also been observed by others. Common issues include illegible demarcations and incomplete questionnaires, making it difficult for healthcare professionals and researchers to accurately use data [16]. Additionally, paper forms need manual scoring, making errors in scoring and data entry possible [17].

Today’s increasingly technological world provides the opportunity to electronically collect PROMs using tablets and smartphones, offering a replacement to the traditional use of paper. The implementation of electronic versions of PROMs can improve data collection, processing and management. Electronic PROMs can help to ensure questions are correctly answered as they do not allow for respondents to create their own answer option and do not allow for interpretation of ambiguous responses [18–20]. Completeness of PROMs may be better in electronic as opposed to paper formats as limits can be placed on data fields, whereby respondents cannot advance to the next question or complete the questionnaire without properly answering all fields [20, 21]. Electronic information technology has been found to reduce the number of data entry errors [19, 22, 23]. Accuracy and efficiency of data collection are also observed as data is automatically calculated, validated, and often transferred to a centralized database so that end users can receive immediate access to the data [18, 24]. Healthcare providers may also observe trends of data over time [25]. A systematic review by Rutherford et al. found that using different modes of administration (including paper versus electronic) did not result in any biases within the patient reported outcome results [26].

Research has shown there is preference towards electronic data capture over paper by healthcare teams due to the improved quality of data capture as well as increased ease of data collection and use [25]. In addition, analysis has shown reductions in cost with the introduction of electronic medical records [27]. Other potential areas of improvement include increased ease of use and decreased time of completion for the patient and/or healthcare team, improvements in patient satisfaction, improved completion rates and reduced physical storage requirements [21, 24, 28]. E-forms can be integrated to assist with pre-visit planning for upcoming clinic visits or facilitate virtual/telemedicine visits [21].

Although previous research indicates that data collected from electronic PROMs are equivalent to their paper form, no research has been conducted using the CHAQ and QoML [29–31]. There have been other studies conducted comparing pediatric electronic PROMs to paper PROMs (such as the Pediatric Quality of Life Inventory (PedsQL) and the Patient-Reported Outcomes Measurement Information System (PROMIS) pediatric measures) but none with the CHAQ and QoML [32, 33]. In addition, it is unclear if the results from Rutherford et al.’s systematic review are transferable to the pediatric rheumatology patient population.

The purpose of this project was to (a) assess whether the implementation of an electronic version of the standardized CHAQ and QoML results in equivalent responses to the paper version; (b) identify potential benefits and barriers associated with electronically capturing PROMs; and (c) gather feedback from patients’ and their caregivers’ regarding their acceptance electronic PROMs, their opinions of integrating its use into regular practice and, as well, gather feedback about their user experience.

## Methods

This was a program evaluation and quality improvement project and a research study which were approved by both the Quality and Risk Management department and the Research Ethics Board, respectively, at The Hospital for Sick Children. A convenience sample of patients/caregivers was enrolled over a one-month period. Patients/caregivers were included in this project if they were able to read and understand English in order to obtain consent, as well as complete the PROMs and survey.

Patients/caregivers scheduled for their upcoming visit with the rheumatology clinic were informed about the project when they received their appointment reminder calls. Those interested in participating were contacted by the project team who informed them that they would be randomized to complete both paper and e-form version of the PROMs and then complete a satisfaction survey. Consent was obtained from patients/caregivers when they arrived at the rheumatology clinic for their scheduled appointment. A screening and enrollment log was maintained to ensure that patients/caregivers were not approached more than once to participate.

### Data collection

An e-form of CHAQ and QoML was created in Research Electronic Data Capture (REDCap) tools hosted at The Hospital for Sick Children [34, 35]. REDCap is a secure, web-based software platform designed to support capture for research studies, providing 1) an intuitive interface for validated data entry; 2) audit trails for tracking data manipulation and export procedures; 3) automated export procedures for seamless data downloads to common statistical packages; and 4) procedures for importing data from external sources [34, 35]. Individuals who consented to participate in this project were asked to complete both the paper and e-form with the order of form completion (paper vs. electronic) determined by a table of random numbers. The time taken to complete each version of the questionnaires was recorded using a timing device. De-identified data from both paper and electronic versions of the form were collected in a database for analysis. Participants were also asked to complete an anonymous satisfaction survey to determine their preferences of form types as well as obtain feedback on their user experience with the e-form platform.

### Data analysis

The rate of questionnaire completion was determined by counting the number of fully completed questionnaires that were returned divided by the total number of questionnaires that were distributed. Scores were compared between the paper and electronic versions of CHAQ and QoML to determine whether differences resulted from the two modes of administration. Equivalence and agreement of the PROMs from the CHAQ and QoML were compared and calculated between the paper form and e-form using ICC estimates and their 95 % confidence intervals. This was calculated by using the R package “irr” and based on a mean rating (k = 2), absolute-agreement, two-way mixed-effects model [36].

Bland-Altman analysis was used to assess bias and the limits of agreement (LoA). Mean scores from the paper form and the e-form were plotted against the difference between these two measurements to obtain Bland–Altman plots [37]. This was done for each of the PROMs. Systematic and random measurement error were assessed with use of the mean difference and the LoA, respectively. The LoA were calculated as the mean difference ± 1.96 the standard deviation (SD) of the mean difference. The LoA describes the interval where 95 % of the difference of the scores measured by the electronic and paper forms are expected to lie [38]. The mean difference is expected to be close to zero with a small interval between the LoA because the methods being compared are expected to be equivalent.

The overall mean and median completion time for the paper and e-form were calculated as well as the mean and median for the new patient and follow-up patient subgroups. Median was used in addition to mean in order to account for outliers and the potential of skew. A time of two and a half minutes (150 s) was added to each paper form completion time to account for the time required for distribution, scoring, data entry, and document management. This number was established prior to the initiation of this project.

Benefits and barriers observed to the implementation of e-form were noted by the quality improvement team. A cost-comparison analysis was conducted by assessing paper form and e-form costs. The cost of each version of the questionnaire was calculated by identifying all resources associated with its creation, distribution, and management. Paper form costs included printing, time taken for staff to manually distribute, collect, score, enter, verify, and deliver the document to the patient’s medical record. It was assumed that an e-form would allow data to be directly accessible electronically without human mediation. E-form costs included the build of the database and the costs of tablets used for this project.

Quantitative and qualitative data were collected from the satisfaction surveys which assessed user experience and collected feedback for improvement. The proportion of participants who preferred the e-form over the paper form, or had no preference, as indicated in their satisfaction survey was calculated. Additionally, participants were asked if the paper and electronic forms were easy to read, understand, and navigate and if the answers were easy to select. The proportion of participants who agreed or disagreed with these prompts were calculated. Participants’ feedback regarding their experience with the e-form and suggestions for user experience improvement were thematically analyzed.

## Results

### Sample

The CHAQ and QoML PROMs are normally distributed in our general clinics, juvenile dermatomyositis (JDM), systemic arthritis and autoinflammatory subspecialty clinics. Therefore, patients seen in our systemic lupus erythematosus (SLE), neonatal lupus erythematosus (NLE), Kawasaki disease, and vasculitis clinics were not included in this study. All patients who were attending these aforementioned clinics were invited to participate, thereby representing the proportion of patients who would usually receive this questionnaire. A convenience sample of 225 clinic patients/caregivers consented to participate. Of the enrolled sample, 29 datasets were excluded due to missing or incomplete data, resulting in a questionnaire completion rate of 87 %. Technical issues with the internet connectivity limited three participants from being able to successfully submit their e-form, whereas we were unable to locate the paper form for 10 participants despite them having submitted an e-form. An additional 11 patients had both uncompleted e-forms and uncompleted paper forms. Finally, five patients were unable to be timed accurately with their paper form as they were interrupted after starting the form (were called to see their healthcare team) and completed the form at a later time. As such, participants who were able to successfully complete both paper and e-forms did so before they saw their attending physician.

A total of 196 participants were included in the project. 21 participants were new patients to the clinic, whereas 175 were follow-up patients. As with our usual clinical practices, we allowed patients and caregivers to decide among themselves who completed the paper and e-form. There was no prescribed eligibility age for patients as secondary factors such as caregiver’s fluency in English and patient’s intellectual/developmental disability influenced this decision. The satisfaction questionnaire was completed by the person who completed the paper and e-forms. Over half (57 %) of the PROMS were completed by the patient alone. 11 % were completed together by the patient and caregiver. The remainder of the PROs were completed by the caregiver.

### Equivalence of Paper form vs. E-Form PROMs assessed by ICCs

The ICC estimates are reported in Table 1. All ICC measures were greater than 0.9 with a *p*-value < 0.001. According to Koo and Li, ICC scores above 0.9 indicate excellent reliability [39]. When we stratified by who completed the PROs (patient versus caregiver) we observed no difference in the agreement of responses (data not shown).

**Table 1:** Intraclass Correlation Coefficients (ICC) Between Paper Forms and E-Forms for PROMs

Patient/Caregiver Reported Outcome Measures	ICC
Quality of my Life (QoML)	
Overall, my life is:	0.910
Considering my health, my life is:	0.910
Childhood Health Assessment Questions (CHAQ)	
CHAQ score	0.966
How would you rate your child’s illness in the past week?	0.904
How much pain do you think your child has had because of his or her illness in the past week?	0.952
Considering all the ways that illness affects your child, rate how your child is doing	0.934

### Agreement and Bias between Paper form vs. E-Form PROMs assessed by Bland-Altman Plots

**Fig. 1:**
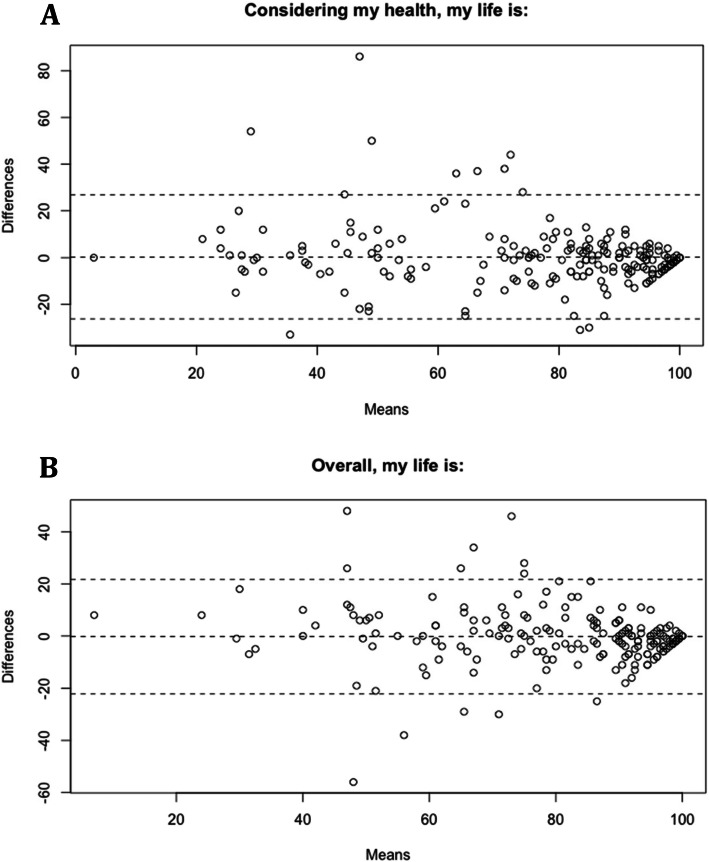
Bland-Altman Plot for QoML Questionnaire. Bland-Altman plot for QoML questionnaire (**a** = “Overall, my life is”, **b** = “Considering my health, my life is”). E-forms were plotted against paper forms. The x-axis is the mean of the two scores entered by the patient on the e-form and the paper form and the y-axis shows the differences between the e-form score and paper form score.

**Fig. 2:**
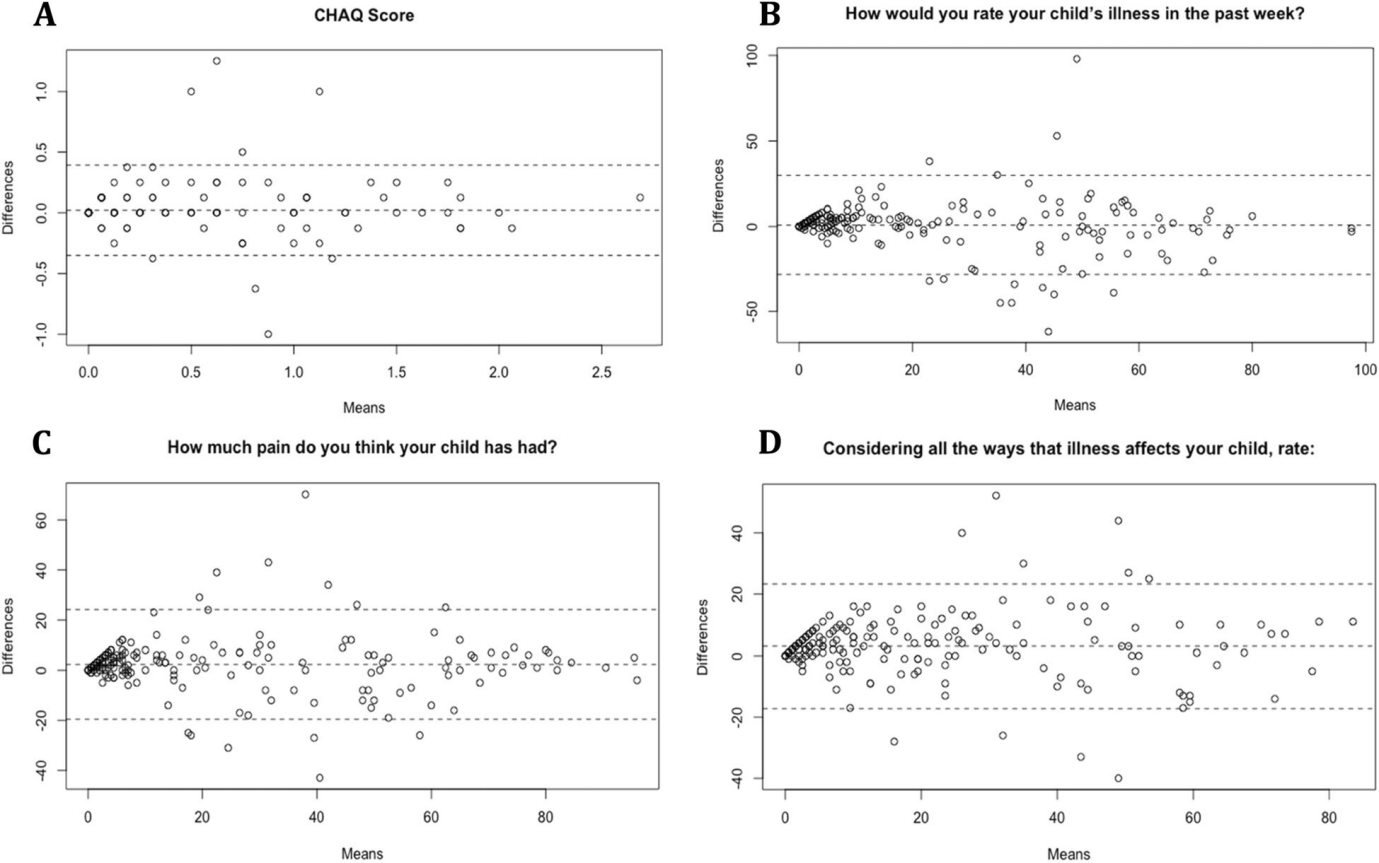
Bland-Altman Plot for CHAQ Questionnaire. Bland-Altman plot for CHAQ questionnaire (**a** = CHAQ Score, **b** = “How would you rate your child’s illness in the past week?”, **c** = “How much pain do you think your child has had because of his or her illness in the past week?”, **d** = “Considering all the ways that illness affects your child, rate how your child is doing”). E-forms were plotted against paper forms. The x-axis is the mean of the two scores entered by the patient on the e-form and the paper form and the y-axis shows the differences between the e-form score and paper form score.

These are shown in Figs. 1 and 2. Figure 1 a and b have data points clustered towards 100 as most of our participants were happy with their quality of life. Figure 2 a, b, c, and d have data points clustered towards 0 as most of our participants did not experience pain that impacts their quality of life. All plots show a certain degree of bias which is listed in the Table 2, along with the LoA.

**Table 2:** Bland-Altman Data Summary for PROMs

Patient/Caregiver Reported Outcomes	Bias^a^	95 % CI of LoA^b^
QoML		
Overall, my life is:	-0.211	26.79 to -26.26
Considering my health, my life is:	0.266	21.75 to -22.17
CHAQ		
CHAQ Score	0.021	0.392 to -0.350
How would you rate your child’s illness in the past week?	0.744	29.78 to -28.29
How much pain do you think your child has had because of his or her illness in the past week?	2.30	24.16 to -19.56
Considering all the ways that illness affects your child, rate how your child is doing:	3.05	23.31 to -17.22

^a^Positive values indicate that on average, paper forms score that many units more than the e-form. Negative values indicate that on average, paper forms score that many units less than the e-form

^b^All scores (except the CHAQ score which is measured out of 3) are measured out of 100

*CI * confidence interval; *LoA* limits of agreement

### Completion Time

Overall, the paper form took longer to complete when 2.5 min were added to the paper form completion time (Table 3). New patients took longer to complete the forms when compared to the follow-up patients. Excluding the processing time, paper forms took less time to complete than the e-forms.

**Table 3:** Mean and Median Completion Time of Paper and Electronic Forms (E-forms) by New and Follow-up Patients

		New (*n* = 21)	Follow-up (*n* = 175)	Overall (*n* = 196)
Paper Form	Mean (seconds)	323.8 (473.8)^a^	255.9 (**405.9**)	263.7 (**413.7**)
	Median (seconds)	302.1 (**452.1**)	207.6 (**357.6)**	221.1 (**371.1**)
E-Form	Mean (seconds)	**519.8**^b^	394.2	407.3
	Median (seconds)	408	293	294.5

^a^Numbers in brackets include two and a half minutes added for manual scoring, verification, and data entry

^b^Bolded numbers indicate the longest time to completion when comparing paper form to e-form

### Observed Benefits and Barriers to electronic PROMs

#### Cost comparison analysis

We identified the costs of all the resources associated with the paper forms and e-forms to accomplish the cost-comparison analysis. The cost per patient for each paper CHAQ/QoML was $1.23 CAD. The overall cost for the e-forms was $500, which included the two electronic tablets used to administer the e-form. Cost savings would be realized after 407 uses of the e-form, which – in our clinic – would take approximately four weeks.

#### Barriers

Barriers to completing the e-form included poor Wi-Fi connectivity in certain areas of the clinic. Patients and caregivers were not able to qualify their answers on the e-form, whereas they could write on the paper form. Sensitivity of the device being used for this project may have decreased the ability of patients/caregivers to select extreme end values (e.g., 0 or 100). Other noted barriers associated with devices were the limited number of devices available, the potential theft of devices, as well as the need to disinfect devices after use.

#### Satisfaction survey results

83 % of respondents indicated that they either preferred the e-form to the paper form or had no preference. One respondent stated: “It was great - easy to use. Easier for my daughter to complete with her arthritis” and another stated: “My daughter usually says ‘Oh no, not again’ when she is handed the paper format. She loved using the tablet format. It is much more user friendly for kids/teens.” Others commented on the e-form’s environmental and potential cost-savings as well.

More than 97 % of participants agreed that both the paper form and e-form were easy to understand and navigate. Approximately 10 % of participants made suggestions to improve the user experience. The respondents commonly reported difficulty selecting responses on e-form. “It was hard to select answers if they were on the extreme end of the sliding scale.” Another common suggestion included making “the text larger, and the select buttons bigger so it is easier to press.”

Three patients/caregivers indicated that they preferred the paper form for varying reasons. “I enjoy writing it with a pen in hand personally” was one reason cited. “The electronic version could go down (not work), and my kids would want to play with it (the tablet) when they see it.” “The paper version was just as fast to complete as the electronic version” were also mentioned by individuals indicating a preference for the paper form.

## Discussion

The two methods of PROMs collection showed excellent agreement, suggesting that the e-form is a reliable and valid replacement for the paper form. This is in-line with previous findings comparing electronic PROMs to paper PROMs [18, 21, 22]. The Bland-Altman plots showed bias that was very close to zero but the LoA were wider than clinically preferred [40]. This is likely due to the difficulty participants experienced using the visual analog scale sliders on the e-form. Some participants reported trouble selecting numbers on the extreme ends of the scale such as “0” or “100”; 0 and 100 are usually the most common responses for the PROMs since many stable patients in a rheumatology clinic are doing well health-wise and have no pain.

As previously hypothesized, the e-form was more efficient than the paper form. Completion and processing time were faster in the electronic groups compared to the paper group after adding the 2.5 min needed for scoring and data management. New patients took more time to complete both forms compared to follow-up patients. This is likely due to the fact that follow-up patients are more familiar with the questions (as they have seen them during their previous visits) and therefore completed the questionnaire faster. However, given that both follow-up and new patients took a longer time to complete the e-form (prior to adding 2.5 min), implies that the novelty of the e-form may not be the only reason associated with longer completion time. We suspect that the extended time was likely due to the recurring difficulty experienced by participants who were attempting to select extreme answers (i.e. zero) on the e-form’s visual analog scale. Furthermore, participants who were familiar with the CHAQ were able to quickly select all of the “without difficulty” answers by drawing a single stroke on the paper form, whereas they were forced to select each multiple choice answer on the e-form.

Our findings are consistent with previous studies stating that electronic forms lead to cost savings after replacing their paper form counterparts [20, 21, 41]. Previous studies also found that e-forms require less time to complete, are more environmentally friendly, and reduce the amount of missing data [18, 21]. If the e-form was built directly into the patient portal of their electronic health record, data could flow seamlessly to the healthcare team. Furthermore, it would avoid any time or cost associated with the need to map data from a separate database to the electronic health record. This would, however, require some resources to set up in the patient’s electronic health record.

Despite the suggested improvements, participants were satisfied with the e-form, which is consistent with other research findings comparing electronic forms to paper forms [42, 43].

To our knowledge, this is the first time that this preference for e-forms was demonstrated with the CHAQ and QoML questionnaires. As many healthcare providers adopt electronic health records (EHRs), the creation of e-forms within the system can assist in efficiently collecting and storing data in one location. Furthermore, in light of the increased number of patients being seen over telemedicine, e-forms can also facilitate the continuity of capturing PROMs during virtual visits.

There were some potential limitations that should be considered when interpreting these findings. First, the use of convenience sampling may have introduced bias into these results. Those patients who were more likely to be seen in the clinic (for more aggressive disease, recurring flare-ups, decreased well-being, etc.) were more likely to be enrolled. However, based on the distribution of new and follow up patients in our sample, we believe we have a good representation of the typical patient population in a rheumatology clinic. Second, the individuals who agreed to participate in this project may have already been more favorably disposed to the e-form and agreed to participate on that basis.

## Conclusions

The e-forms of CHAQ and QoML obtain equivalent responses as the paper forms. Multiple benefits are associated with implementation of an e-form including efficiency, cost-savings, and patient satisfaction. We plan to implement the suggested improvements and incorporate the finalized e-form into our clinic as standard practice.

## Data Availability

The datasets used and/or analyzed during the current study are available from the corresponding author on reasonable request.

## Abbreviations

ADL: Activities of daily living
CI: Confidence interval
CHAQ: Childhood Health Assessment Questionnaire
e-form: Electronic form
EHR: Electronic health record
ICC: Intraclass correlation coefficient
JDM: Juvenile dermatomyositis
LoA: Limits of agreement
NLE: Neonatal lupus erythematosus
PedsQL: Pediatric Quality of Life Inventory
PROMIS: Patient-Reported Outcomes Measurement Information System
PROMs: Patient reported outcome measures
QoML: Quality of My Life
REDCap: Research Electronic Data Capture
SickKids: The Hospital for Sick Children
SLE: Systemic lupus erythematosus
VAS: Visual analog scale

## References

[1] Dawson J, Doll H, Fitzpatrick R, Jenkinson C, Carr AJ (2010). The routine use of patient reported outcome measures in healthcare settings. BMJ.

[2] U. S. Food and Drug Administration. Guidance for industry. Patient-reported outcome measures: Use in medical product development to support labeling claims [Internet]. Clinical/Medical Federal Register. 2009. Available from: https://www.fda.gov/regulatory-information/search-fda-guidance-documents/patient-reported-outcome-measures-use-medical-product-development-support-labeling-claims.

[3] Black N (2013). Patient reported outcome measures could help transform healthcare. BMJ.

[4] Santana MJ, Feeny D (2014). Framework to assess the effects of using patient-reported outcome measures in chronic care management. Qual Life Res.

[5] Luca NJC, Feldman BM (2014). Health outcomes of pediatric rheumatic diseases. Best Pract Res Clin Rheumatol.

[6] Shoop-Worrall SJW, Hyrich KL, Verstappen SMM, Sergeant JC, Baildam E, Chieng A (2020). Comparing proxy, adolescent, and adult assessments of functional ability in adolescents with juvenile idiopathic arthritis. Arthritis Care Res.

[7] Gong GWK, Young NL, Dempster H, Porepa M, Feldman BM (2007). The quality of my life questionnaire: The minimal clinically important difference for pediatric rheumatology patients. J Rheumatol.

[8] Singh G, Athreya BH, Fries JF, Goldsmith DP (1994). Measurement of health status in children with juvenile rheumatoid arthritis. Arthritis Rheum.

[9] Meiorin S, Pistorio A, Ravelli A, Iusan SM, Filocamo G, Trail L (2008). Validation of the childhood health assessment questionnaire in active juvenile systemic lupus erythematosus. Arthritis Care Res.

[10] Goycochea-Robles MV, Garduño-Espinosa J, Vilchis-Guizar E, Ortiz-Alvarez O, Burgos-Vargas R. Validation of a Spanish version of the Childhood Health Assessment Questionnaire. J Rheumatol. 1997 Nov;24(11):2242–5. 9375891

[11] Ouwerkerk JW, van Pelt PA, Takken T, Helders PJ, van der Net J. Evaluating score distributions in the revised Dutch version of the Childhood Health Assessment Questionnaire. Pediatr Rheumatol. 2008 Sep;6:14. 10.1186/1546-0096-6-14 PMC2546387 18786245

[12] Foeldvari I, Ruperto N, Dressler F, Häfner R, Küster RM, Michels H, et al. The German version of the Childhood Health Assessment Questionnaire (CHAQ) and the Child Health Questionnaire (CHQ). Clin Exp Rheumatol. 2001;19(4 SUPPL. 23):19–22. 11510335

[13] Dempster H, Porepa M, Young N, Feldman BM (2001). The clinical meaning of functional outcome scores in children with juvenile arthritis. Arthritis Rheum.

[14] Oen K, Guzman J, Dufault B, Tucker LB, Shiff NJ, Duffy KW (2018). Health-Related Quality of Life in an inception cohort of children with juvenile idiopathic arthritis: A longitudinal analysis. Arthritis Care Res.

[15] Feldman BM, Grundland B, McCullough L, Wright V (2000). Distinction of quality of life, health related quality of life, and health status in children referred for rheumatologic care. J Rheumatol.

[16] Dale O, Hagen KB (2007). Despite technical problems personal digital assistants outperform pen and paper when collecting patient diary data. J Clin Epidemiol.

[17] Kaushal R, Shojania KG, Bates DW (2003). Effects of computerized physician order entry and clinical decision support systems on medication safety: A systematic review. Arch Intern Med.

[18] VanDenKerkhof EG, Goldstein DH, Blaine WC, Rimmer MJ (2005). A comparison of paper with electronic patient-completed questionnaires in a clinic. Anesth Analg.

[19] Bates DW, Cohen M, Leape LL, Overhage JM, Shabot MM, Sheridan T (2001). Reducing the frequency of errors in medicine using information technology. J Am Med Informatics Assoc.

[20] Galliher JM, Stewart TV, Pathak PK, Werner JJ, Dickinson LM, Hickner JM (2008). Data collection outcomes comparing paper forms with PDA forms in an office-based patient survey. Ann Fam Med.

[21] Coons SJ, Eremenco S, Lundy JJ, O’Donohoe P, O’Gorman H, Malizia W (2015). Capturing patient-reported outcome (PRO) data electronically: the past present, and promise of ePRO measurement in clinical trials. Patient.

[22] Bernhard J, Cella DF, Coates AS, Fallowfield L, Ganz PA, Moinpour CM (1998). Missing quality of life data in cancer clinical trials: Serious problems and challenges. Stat Med.

[23] Agrawal A (2009). Medication errors: Prevention using information technology systems. Br J Clin Pharmacol.

[24] Hernar I, Graue M, Richards D, Strandberg RB, Nilsen RM, Tell GS (2019). Electronic capturing of patient-reported outcome measures on a touchscreen computer in clinical diabetes practice (the DiaPROM trial): A feasibility study. Pilot Feasibility Stud.

[25] Le Jeannic A, Quelen C, Alberti C, Durand-Zaleski I (2014). Comparison of two data collection processes in clinical studies: Electronic and paper case report forms. BMC Med Res Methodol.

[26] Rutherford C, Costa D, Mercieca-Bebber R, Rice H, Gabb L, King M (2016). Mode of administration does not cause bias in patient-reported outcome results: a meta-analysis. Qual Life Res.

[27] Greenlaw C, Brown-Welty S (2009). A comparison of web-based and paper-based survey methods: Testing assumptions of survey mode and response cost. Eval Rev.

[28] Campbell N, Ali F, Finlay AY, Salek SS (2015). Equivalence of electronic and paper-based patient-reported outcome measures. Qual Life Res.

[29] Ashley L, Keding A, Brown J, Velikova G, Wright P (2013). Score equivalence of electronic and paper versions of the Social Difficulties Inventory (SDI-21): A randomised crossover trial in cancer patients. Qual Life Res.

[30] Coons SJ, Gwaltney CJ, Hays RD, Lundy JJ, Sloan JA, Revicki DA (2009). Recommendations on evidence needed to support measurement equivalence between electronic and paper-based patient-reported outcome (PRO) measures: ISPOR ePRO good research practices task force report. Value Heal.

[31] Gwaltney CJ, Shields AL, Shiffman S (2008). Equivalence of electronic and paper-and-pencil administration of patient-reported outcome measures: A meta-analytic review. Value Heal.

[32] Hinds PS, Nuss SL, Ruccione KS, Withycombe JS, Jacobs S, Deluca H, et al. PROMIS pediatric measures in pediatric oncology: Valid and clinically feasible indicators of patient-reported outcomes. Pediatr Blood Cancer. 2013 Mar;60(3):402–8. 10.1002/pbc.24233 22829446

[33] Vinney LA, Grade JD, Connor NP (2012). Feasibility of using a handheld electronic device for the collection of patient reported outcomes data from children. J Commun Disord.

[34] Harris PA, Taylor R, Thielke R, Payne J, Gonzalez N, Conde JG (2009). Research electronic data capture (REDCap)-A metadata-driven methodology and workflow process for providing translational research informatics support. J Biomed Inform.

[35] Harris PA, Taylor R, Minor BL, Elliott V, Fernandez M, O’Neal L, et al. The REDCap consortium: Building an international community of software platform partners. J Biomed Inform. 2019;95(103208). 10.1016/j.jbi.2019.103208 PMC7254481 31078660

[36] Garner M, Lemon J, Fellows I, Singh P. irr: Various Coefficients of Interrater Reliability and Agreement. R package version 0.83. [Internet]. 2010. Available from: https://rdrr.io/cran/irr/.

[37] Bland MJ, Altman DG (1986). Statistical methods for assessing agreement between two methods of clinical measurement. Lancet.

[38] Bland JM, Altman DG (1999). Measuring agreement in method comparison studies. Stat Methods Med Res.

[39] Koo TK, Li MY (2016). A Guideline of Selecting and Reporting Intraclass Correlation Coefficients for Reliability Research. J Chiropr Med.

[40] Giavarina D (2015). Understanding Bland Altman analysis. Biochem Medica.

[41] Jose N, Langel K. ePRO vs. paper [Internet]. Applied Clinical Trials Online. 2010 [cited 2020 May 1]. Available from: http://www.appliedclinicaltrialsonline.com/epro-vs-paper.

[42] Recinos PF, Dunphy CJ, Thompson N, Schuschu J, Urchek JL, Katzan IL (2017). Patient satisfaction with collection of patient-reported outcome measures in routine care. Adv Ther.

[43] Schamber EM, Takemoto SK, Chenok KE, Bozic KJ (2013). Barriers to completion of patient reported outcome measures. J Arthroplasty.

